# *Chlamydia trachomatis* Vaccine Research through the Years

**DOI:** 10.1155/2011/963513

**Published:** 2011-06-26

**Authors:** Katelijn Schautteet, Evelien De Clercq, Daisy Vanrompay

**Affiliations:** Department of Molecular Biotechnology, Faculty of Bioscience Engineering, Ghent University, Coupure Links 653, 9000 Ghent, Belgium

## Abstract

*Chlamydia trachomatis* is a Gram-negative obligate intracellular bacterium. It is the leading cause of bacterial sexual transmitted infections (STIs). World Health Organization figures estimated that over 90 million new cases of genital *C. trachomatis* infections occur worldwide each year. A vaccination program is considered to be the best approach to reduce the prevalence of *C. trachomatis* infections, as it would be much cheaper and have a greater impact on controlling *C. trachomatis* infections worldwide rather than a screening program or treating infections with antibiotics. Currently, there are no vaccines available which effectively protect against a *C. trachomatis* genital infection despite the many efforts that have been made throughout the years. In this paper, the many attempts to develop a protective vaccine against a genital *C. trachomatis* infection will be reviewed.

## 1. Introduction

*Chlamydia trachomatis* is a Gram-negative obligate intracellular bacterium. It is the leading cause of bacterial sexually transmitted disease in both developed and developing countries with more than 90 million new cases of genital *C. trachomatis *infections occurring each year [1]. In the past years, an increase in the number of STIs and in particular of *C. trachomatis *infections has been observed in many, if not all, European countries [2]. This increase might be attributed to changes in attitudes, increased awareness of healthcare workers, and improved diagnostics. 

In the genital tract, infection with *C. trachomatis *is propagated within the single cell columnar layer of the epithelium in the urethra of men and the endocervix of women. Within the epithelial cells, *C. trachomatis *undergoes a unique biphasic developmental cycle consisting of an infectious, but metabolically inert, elementary body (EB) and a noninfectious, but metabolically active, reticulate body (RB). After completion of the developmental cycle, the EBs are released and infect neighboring epithelial cells, thereby spreading the infection. 

Infection can result in acute inflammation characterized by redness, edema, and mucosal discharge and is diagnosed clinically as mucopurulent cervicitis in women and non-gonococcal urethritis in men [3, 4]. In women, infection can manifest as abnormal vaginal discharge and/or postcoital bleeding, while the infection is limited to the lower genital tract and irregular uterine bleeding and/or pelvic discomfort once the infection ascends to the upper genital tract [4]. Symptoms in males are generally limited to dysuria and moderate clear to whitish discharge [4]. While these symptoms signify an infection, the absence of such symptoms does not necessarily indicate the absence of infection. Up to 75% of women and 50% of men infected with *C. trachomatis* are asymptomatic [5, 6], and these infected people do not seek medical attention. If the infection remains untreated, it often results in pelvic inflammatory disease (PID), tubal scarring, ectopic pregnancy, and chronic pelvic pain in women which could lead to infertility, epididymitis in men, and infant pneumonia in children [7–10]. 

Although very effective antimicrobial therapy is available, a vaccination program is considered to be the best approach to reduce the prevalence of *C. trachomatis* infections. It would be much cheaper and have a greater impact on controlling *C. trachomatis* infections worldwide than a screening program or treating infections with antibiotics. Long-term induced immunity against STIs such as *C. trachomatis* would be preferable. However, since STIs have the highest incidence at the reproductive age, even short- to medium-term immunity would be of great benefit. Therefore, a *C. trachomatis* vaccine protecting at least women in their fertile period against complications would be a valuable tool in achieving a higher level of public health. Currently, there are no vaccines available against a *C. trachomatis* genital infection despite the many efforts that have been made throughout the years to develop a protective *C. trachomatis* vaccine. In this paper the many attempts to develop a protective vaccine against a genital *C. trachomatis* infection will be reviewed.

## 2. *Chlamydia muridarum* versus *C. trachomatis* Mouse Models

The most used animal model to study *C. trachomatis* female tract infections is the mouse model. The mouse is susceptible to *C. muridarum *mouse pneumonitis (MoPn), formerly known as the mouse biovar of *C. trachomatis*, and to human genital tract isolates of *C. trachomatis*. The genomes of these species share remarkable similarities in the content and order of genes and in the presence of putative virulence factors [11]. An important difference between the species is the absence and presence of a tryptophan operon in the genome of *C. trachomatis* and *C. muridarum*, respectively [12, 13]. Consequently, these biovars have differential sensitivity to IFN-*γ*, a cytokine which plays an important role in the early clearance of chlamydia from the genital tract [14]. Most likely the *C. muridarum* and the *C. trachomatis* strain will also differ in response to other cytokines. 

There are also significant differences in virulence characteristics among both biovars. In contrast to human isolates, *C. muridarum* is able to cause severe upper genital tract pathology and a high incidence of infertility after a single infection in mice [15]. In addition, the developmental cycle of *C. muridarum* is more rapid, its duration being approximately half that of human strains, and the strain is more prolific. *Chlamydia muridarum* can infect mice of various strains nearly equally, while infection of mice with *C. trachomatis* is highly dependable on the mouse strain. Overall, lower shedding and minimal to moderate inflammation can be noticed in mice infected with *C. trachomatis*. Furthermore, postinfection sequelae are less common. This is in accordance to the fact that upper genital tract progression followed by pathology, usually resulting from multiple infections, is only seen in a small percentage of women [16]. 

In contrast to *C. muridarum* infection, *C. trachomatis* infection was unaltered in the absence of CD4^+^ T cells. Mice infected with *C. trachomatis* developed protective immunity to rechallenge, but unlike *C. muridarum* infection, optimum resistance required multiple infectious challenges despite the generation of adaptive serum and local chlamydial specific immune responses. Thus, understanding the chlamydial pathogenic and host immunologic factors that result in a diminished protective role for CD4^+^ T cells in *C. trachomatis* murine infection might lead to new insights important to human immunity and vaccine development [17]. It has been demonstrated that strong adaptive immune responses are generated when mice are infected with *C. trachomatis* serovars [3, 18, 19], but it has also been shown that these infections in mice can resolve in the absence of adaptive immunity, suggesting that innate immune responses alone can resolve infection [13]. This is not necessarily a reason to invalidate the use of *C. trachomatis* serovars in murine studies of genital tract infection or in vaccine development. It could be that the rather mild infection seen in murine studies utilizing human *C. trachomatis* biovars may replicate some aspects of human infection. In order to resolve the murine *C. muridarum* genital infection and to protect against reinfection, adaptive immune responses are absolutely indispensable. 

As there are considerable differences between the *C. muridarum* and the *C. trachomatis* murine model, it is difficult to make direct comparisons. In order to understand the pathogenesis of human chlamydial infections completely, it is absolutely necessary to thoroughly investigate chlamydial infection in its natural human host [20].

## 3. Protective Immune Responses to *C. trachomatis*

Information on the immune mechanisms of clearance of infection and resistance to reinfection has been provided in particular by mouse models of genital infection. T cells, especially major histocompatibility complex (MHC) class II-restricted CD4^+^ T cells, are required for protective immunity [21–24]. MHC class I-restricted CD8^+^ T cells, on the other hand, are not necessary for infection resolution or immunity to reinfection [21–24]. The protective role of antibody is less easily discernible than that of the cellular response, but important to vaccine development, it is as protective as CD4^+^ T cells in immunity to reinfection [22, 25]. Furthermore, Th1 cytokines, specifically IFN-*γ* and interleukin-12 (IL-12), are essential to induce a protective response [13, 26, 27]. In women, CD4^+^ T cells are indeed recruited to the cervix during active infection; however, CD8^+^ and dentritic cells are also recruited, and the relative proportions of these cells may be situational. Different studies involving women have confirmed that local Th1 cytokines, mainly IFN-*γ*, are associated with *C. trachomatis* infection (reviewed by [28]) although these studies have not been able to determine which specific responses lead to infection resolution versus persistence [29–31]. Serum and genital mucosal IgG and IgA antibodies to specific *C. trachomatis* proteins and to chlamydial EBs are usually detected during active infection in women [32–34]. These antibody responses in humans infected with *C. trachomatis*, including those measured in endocervical secretions, have not been found to correlate with protective immunity but appear to be markers of prior infection. 

When developing a vaccine against genital *C. trachomatis* infections, it is important to take into account the unique properties of the genital tract. This mucosal site is unique among mucosal effector tissues, as it lacks organized lymphatics which can result in a delayed systemic response relative to other sites [35]. Furthermore, the female genital tract is also subjected to hormonal regulation, and the effectiveness of intravaginal vaccination has been shown to be influenced by the phase of the menstrual cycle [35–37]. The immunological characteristics of the genital tract and the tropism of chlamydia for mucosal epithelial cells show that a *C. trachomatis* vaccine has to induce both mucosal and systemic protective responses.

## 4. Whole Organism Vaccines—First-Generation *C. trachomatis* Vaccines

Initial attempts to develop an effective vaccine for controlling both animal and human chlamydial infections began with the use of inactivated or live, attenuated whole organism preparations in the 1950s. These vaccines can offer a degree of protection but are far from ideal. Common problems are the cost and the complexity of production, the requirement for cold storage, the presence of antigens which can induce autoimmunity or immunopathology, and the limited efficacy in neonates with high levels of maternal antibodies [38]. 

### 4.1. Live Attenuated Organisms

The first vaccines that were used against *Chlamydiaceae* were live vaccines. With this method of immunization, attenuated or modified living chlamydial organisms were used. The development of attenuated strains usually happens by a number of passages of the wild-type strain in different types of cell cultures or by chemical mutagenesis. Due to the passages, one or more mutations could arise, resulting in a nonvirulent attenuated strain. Live attenuated vaccines can elicit humoral and cellular immunity, because they replicate in a manner analogous to the target pathogen, promoting the processing and presentation of antigens in a way that is most similar to the natural infection [39]. On the other hand, they can also revert to the virulent wild-type strain resulting in disease or persistent infection. Whole-organism vaccination is unlikely to be attempted in the near future, because there is a risk of immunopathology, the large-scale production of pure chlamydiae is extremely difficult [40] and because of the possible spread of live *Chlamydiaceae* in the environment [41].

In the 1960s, unsuccessful vaccination trials with live attenuated vaccines against trachoma were performed in humans and primates [42]. Four decades later, several authors have explored the possibility to vaccinate with live attenuated bacteria against genital *C. trachomatis* infection.

Peterson et al. [43] immunized mice intranasally or intraperitoneally with viable *C. trachomatis*, serovar E. Mice immunized intranasally with live *C. trachomatis* exhibited significant protection upon a vaginal infection, while intraperitoneally immunized mice did not. However, the protection was not complete. Su et al. [44] performed an experiment in mice to investigate the ability of a live attenuated *C. trachomatis* vaccine to prevent genital infection. Mice were treated with a subchlamydiacidal concentration of oxytetracycline following vaginal infection. Results showed that a self-limiting subclinical infection of the murine genital tract with *C. trachomatis* is as efficient as a clinically apparent acute infection in generating a protective anti-chlamydial immune response. Based on these results, the authors concluded that a live attenuated vaccine would be useful for the prevention of chlamydial STIs. Recently, Olivares-Zavaleta et al. [45] evaluated the protective immunity of the attenuated *C. trachomatis* L2 (25667R) strain in a murine model. They concluded that intravaginal vaccination with the live-attenuated strain L2 is safe, induces a systemic antibody and a CD4^+^  Th1-based immune response, but its protective efficacy is limited to reducing chlamydial burden at early time periods after-infection. 

Recently, Yu et al. [46] vaccinated mice intranasally with live *C. muridarum* with or without CpG-containing oligodeoxynucleotide 1862. Immunization elicited widely disparate levels of protective immunity to genital tract challenge. Protection was correlated with the frequency of multifunctional T cells coexpressing IFN-*γ* and TNF-*α* with or without IL-2. These results suggest that IFN-*γ* producing CD4^+^ T cells that highly coexpress TNF- *α* may be the optimal effector cells for protective immunity.

In view of the safety aspects (possible return to the virulent wild type strain) and the risk for immunopathological damage, it seems unlikely that a live attenuated *C. trachomatis* vaccine will be allowed in humans.

### 4.2. Inactivated or Killed Organisms

Because live vaccines are not always safe or available, research switched to the use of killed or inactivated organisms. Inactivation was done by heat or chemical treatment. Compared to live organisms, inactivated or killed vaccines also have some disadvantages. They may contain undesirable components like bacterial endotoxins, that can cause detrimental side effects, or nonprotective components that may reduce the degree of protection that is required. Their major disadvantage is that they are not able to replicate anymore, which stresses the need to revaccinate and to use adjuvants. Another consequence of their inability to replicate is that they are poor inducers of cell-mediated immunity although they can induce and adequate level of humoral immunity [38]. Because a strong cell-mediated immunity is needed for clearance of chlamydial infections, inactivated or killed organisms seem to be less suitable for vaccine development against *Chlamydiaceae*. 

Studies on inactivated or killed organism vaccines against genital *C. trachomatis* infection are rare. In this study, Peterson et al. [43] failed to elicit a protective response to a vaginal *C. trachomatis* infection in mice immunized intranasally and intraperitoneally with 1 × 10^6^ UV inactivated inclusion forming units of *C. trachomatis* serovar E.

## 5. Subunit Vaccines—Second-Generation *C. trachomatis* Vaccines

In order to avoid harmful effect of the preparations containing the whole organism, it was proposed that a subunit vaccine was needed. Subunit vaccines are safer, they cannot revert to a virulent form, and undesirable antigens, which can induce immunopathology or inflammatory damage, can be avoided [47]. Vaccine candidate antigens, or parts of antigens, may be represented as purified proteins, recombinant proteins or as synthetic proteins [48]. But subunit vaccines have also some disadvantages. Like inactivated vaccines, they are poor inducers of cell-mediated immunity [38], which is very important in the defense against chlamydial infections. Furthermore, the use of adjuvants is being recommended. 

### 5.1. Purified MOMP and COMC Preparations

Following the identification of the major outer membrane protein (MOMP) as the structurally and immunologically dominant protein in the chlamydial outer membrane [49], vaccine research mainly focused on this protein. Some results were encouraging while others rather disappointing. Pal et al. [50] found that a chlamydial outer membrane complex (COMC) preparation of *C. muridarum* could induce significantly protective immunity in mice against a genital challenge, while purified MOMP preparations could not. Some years later, the same research group immunized mice with a purified and refolded preparation of the *C. muridarum* MOMP in combination with Freund's adjuvants. A significant level of protection was conferred in the vaccinated mice against a genital challenge [51]. Cheng et al. [52] demonstrated the protective potential of native MOMP of a *C. muridarum* serovar in combination with novel adjuvants, the nontoxic subunit B of cholera toxin (CTB-CpG). Immunization elicited a significant antigen-specific antibody and cell-mediated immune response as well as protection against a pulmonary challenge with *C. muridarum*. Cunningham et al. [53] could demonstrate that immunization of mice with purified *C. muridarum* MOMP could induce neutralizing antibodies which leaded to reduced numbers of infected mice. Surprisingly, these antibodies also accelerated the development of severe oviduct pathology. Therefore, it is important to keep in mind that immunity can potentially induce pathology and this should be considered when designing vaccines.

Igietseme and Murdin [54] prepared a MOMP-ISCOM vaccine based on MOMP extracted from *C. trachomatis* serovar D. This vaccine was able to produce a Th1 antigen-specific immune response, and immunized mice cleared a vaginal infection within one week.

From these studies, it is clear that some preparations can induce more protection than others. This is probably due to the difference in extraction method which can influence the preservation of conformational MOMP epitopes, necessary for protection. Although vaccination with refolded, purified MOMP preparations have been reasonable successful, the major drawbacks of these vaccines are that they are very expensive and there are problems to grow chlamydia in bulk, which renders these kinds of vaccines commercially non-viable [42].

### 5.2. Recombinant Proteins

Nowadays, it is possible to produce high amounts of bacterial proteins by recombinant DNA technology which is cheaper and more cost effective. The genes, coding for protective antigens, will be expressed in prokaryotic or eukaryotic cells that will produce the desired recombinant protein. For chlamydial vaccines, recombinant MOMP (rMOMP) is generally used. However, the expression of full-length rMOMP in prokaryotic expression systems is generally toxic, and it is also difficult to produce rMOMP in a native form with intact, conformationally relevant epitopes [55]. Moreover, the chlamydial MOMP is glycosylated [56, 57]. 

Different attempts were made to elicit protection against a *C. trachomatis* infection by rMOMP vaccination. Transcutaneous immunization with MOMP in combination with the cholera toxin and CpG oligodeoxynucleotides elicits IgG and IgA antibody response in the vaginal and cervical lavage fluid and an IgG antibody response in the serum. Furthermore, IFN-*γ* secreting T cells were activated in the draining lymph nodes. The immunization protocol resulted in enhanced clearance of *C. muridarum* following intravaginal challenge of mice [58]. Pal et al. [59] demonstrated that immunisation with purified *C. muridarum* MOMP, co-administered with *Borrelia burgdorferi* Outer surface protein (Osp) A as adjuvant, can induce significant protection in mice against a *C. muridarum* genital infection. Sun et al. [60] compared vaccines based on recombinant (rMOMP) and native MOMP (nMOMP). The recombinant preparation based on *C. muridarum* MOMP can elicit a protective immune response in mice against an intranasal challenge. However, the degree of protection obtained with the rMOMP was not as robust as that achieved with an nMOMP preparation indicating that the structural conformation of the MOMP is important for inducing protection. Hickey et al. [61] showed that transcutaneous immunization of mice with rMOMP incorporated in lipid C, induces partial protection of both the respiratory and genital mucosae against challenge with *C. muridarum*. The efficacy of a recombinant vaccine is not only defined by the protein that is used but also by the administration routes. It has been proven that a combined systemic and mucosal vaccination with rMOMP provides better protection against a challenge with *C. muridarum* than either systemic or mucosal immunization alone [62]. Systemic immunization of mice with rMOMP from *C. trachomatis* could reduce the number of animals developing severe salpingitis but failed to reduce chlamydial colonization of the lower genital tract. Mice, immunized with rMOMP directly into the Peyer's patches (to stimulate mucosal immunity), shed fewer chlamydiae from the vagina, but showed little reduction in oviduct damage. Furthermore, the number of animals developing severe salpingitis could not be reduced. Although in both cases specific IgG and IgA antibody responses could be observed, they could not completely protect the mice [63].

Although most recombinant vaccines are based on MOMP, other proteins can also be viable vaccine candidates. In 2007, a novel vaccination strategy using a secreted protein, chlamydial protease-like activity factor (CPAF) was developed by Murthy et al. [64]. Intranasal immunization using recombinant CPAF (rCPAF) accompanied by interleukin-12 (IL-12) was used to assess the protective immunity against genital *C. muridarum* infection in BALB/c mice. rCPAF + IL-12-vaccinated mice displayed significantly reduced bacterial shedding upon chlamydial challenge and accelerated resolution of infection compared to mock-immunized animals. Moreover, rCPAF + IL-12-immunized animals exhibited protection against pathological consequences of chlamydial infection. These results demonstrate for the first time that a secreted chlamydial protein, CPAF, is a viable vaccine candidate that should be considered for induction of efficacious, antichlamydial immunity. The chlamydial proteins OmcB and rl16 have been identified as human B and T cell targets during chlamydial infections in humans [65, 66]. Vaccination of mice with a fusion protein (CTH1) composed of those two antigens promoted a CD4^+^ T-cell dependent protective response but lacks a CD4 independent protective mechanism for complete protection [67].

### 5.3. Synthetic Peptides

Today, computer-based methods to predict antigenic domains or epitopes are available. Synthetic production of these epitopes makes it possible to produce synthetic peptides which correspond with the important immunogenic domains on the antigens. On the other hand, we have to take into account that a lot of antigenic determinants need conformational or three-dimensional structures, like in the complete protein, to elicit an immune response. 

Studies with MOMP peptides and oligopeptide vaccines showed variable results with maximum partial protection. Preliminary studies in mice indicated that intradermal injection of a peptide from a conserved region of the MOMP of *C. trachomatis*, conferred some protection against the development of salpingitis [68]. In contrast to these findings, Su et al. [69] found that parenteral immunization of mice with an alum-adsorbed synthetic oligopeptide of the *C. trachomatis *MOMP, was ineffective in preventing chlamydial genital tract infection although mice produced high levels of antichlamydial serum IgG neutralizing antibodies. Therefore, DNA vaccination which induces both humoral and cellular immune responses can be an alternative method to protect animals from chlamydial infections.

## 6. DNA Vaccines—Third-Generation *C. trachomatis* Vaccines

DNA immunization represents a novel approach to vaccine and immunotherapeutic development. Injection of plasmid DNA encoding a foreign gene of interest can result in the subsequent expression of the foreign gene product and the induction of an immune response within the host. DNA vaccines have a number of advantages when compared with alternative vaccination strategies [70]. They encode multiple immunogenic epitopes and evoke both humoral and cell-mediated immune responses. The immunogenic epitopes are presented to the immune system in their native form. Therefore, DNA vaccines exhibit the advantages of attenuated vaccines without the safety problems associated with the *in vivo* replication and possible reversion to a virulent form. Due to the endogenous production of the antigen, a more balanced Th1/Th2 like immune response is elicited [71]. Plasmid vectors can be rapidly constructed and easily tested. Large-scale manufacturing procedures are available and the DNA can be easily and inexpensively purified to homogeneity, resulting in lower costs to develop and manufacture this type of vaccine [42, 72]. This makes this strategy applicable as a human vaccine approach in underdeveloped countries and as a veterinary vaccine strategy, where the cost per dose is of major economic concern. In addition, DNA is more thermostable than vaccine strategies which require a cold chain for storage [73], and it should exhibit a longer shelf-life because of the improved stability. The production of combination vaccines employing DNA is also simplified. DNA also allows a more simplified and effective quality control process that provides additional cost benefits.

In addition, there are some concerns and potential disadvantages of DNA vaccines. Firstly, the DNA could possibly integrate into the host chromosome. This has not been proven yet, and it is thought that the chance that this will happen is lower than the spontaneous mutation frequency [74]. A second concern of DNA vaccination is the possibility of generating antibodies to DNA. Immune responses to DNA occur in autoimmune diseases, and the possibility exists that bacterial DNA injection could induce an immune response that might cross-react with host DNA [70]. Thirdly, long-term expression of injected DNA into muscle cells may have an effect on immune responses to subsequent vaccination with different DNA, and the immune responses to protective epitopes associated with this second immunization can be compromised. The fourth disadvantage is that DNA vaccination strategies are unsuccessful when evaluating non-protein-based antigens, such as bacterial polysaccharides and lipids [70]. Other possible disadvantages are the low transfection and expression efficiency of DNA vaccines, certainly in large animals and humans [75]. However, by using various combinations of delivery systems and different adjuvants, the immune response can be enhanced. In the past, different studies have evaluated the protective potential of DNA vaccines against chlamydial infections. 

### 6.1. *C. trachomatis* DNA Vaccination

The first attempt to generate an MOMP-based DNA vaccine against a genital chlamydial challenge was disappointing [76]. This vaccine encoded the MOMP gene of *C. muridarum*. Only modest immune response was elicited, but no protection could be established against infection or disease. Because DNA immunization alone did not generate immune responses or protection to the same extent as those induced by using live organisms, combinational vaccines were evaluated. DNA priming followed by boosting with immune-stimulating complexes (ISCOM) of MOMP protein (MOMP ISCOM) in mice resulted in higher protection when compared to mice given MOMP ISCOM immunization alone [77]. In 2010 and 2011, Schautteet et al. [78, 79] studied the ability of a DNA vaccine based on *C. trachomatis* MOMP to protect against genital *C. trachomatis* infection in a recently developed pig model [80]. When administrating the vaccine to the vaginal mucosa, a cellular immune response was induced which elicited significant protection in pigs. The infection could not be cleared completely [79]. When the DNA vaccine was administered combined to the nasal and vaginal mucosa of the pig, both cellular and humoral immune responses were induced which contributed to the significant protection of pigs against a genital *C. trachomatis* infection [78]. 

Since a couple of years, other genes than *ompA* were evaluated for their potential as vaccine candidates. DNA immunization with the *pgp3* gene of *C. trachomatis *could inhibit the spread of the infection from the lower to the upper genital tract [81]. The *pgp3* gene encodes a 28 kDa polypeptide found on the pCT plasmid of *C. trachomatis* which may provide a function related to chlamydial cell physiology [82]. Ifere et al. [83] developed a DNA vaccine composed of MOMP and the porin B protein (PorB) of *C. trachomatis*. A recombinant *Vibrio Cholerae* ghost (rVCG) was used as carrier and delivery system. Significant higher levels of Th1 response and secretory IgA and IgG2a were induced by immunization. Furthermore, all animals which were immunized with the multisubunit vaccine completely resolved the infection two weeks after challenge. In 2008, a pORF5 DNA vaccine was evaluated for its protective immunity in a mouse model of genital chlamydial infection. The vaccinated mice displayed significantly reduced bacterial shedding upon chlamydial challenge and an accelerated resolution of the infection. Furthermore, the immunized mice also exhibited protection against pathological consequences of chlamydial infection. These results demonstrate the potential of the pORF5 DNA vaccine to elicit protective immunity against a genital chlamydial challenge [84].

## 7. Impact of a *C. trachomatis* Vaccine

Recently, a mathematical model has been developed that simulates transmission in a heterosexual population by linking the within-host biology of susceptibility and the chlamydia-infected individuals to their sexual behavior and partnership dynamics [85]. The model tracks the infection time course, disease progression, and dynamic infectiousness of infected individuals and the transmission to others. The authors have demonstrated that if a fully protective vaccine is available, and this will be administered to adolescents before their sexual debut, epidemics of chlamydia infection could be eradicated within 20 years. Furthermore, it is likely that targeting 100% of one sex (females) will have a greater epidemiological impact than administering vaccines to 50% of both sexes. If lifelong sterilizing immunity cannot be achieved, a chlamydia vaccine should be effective for at least 10 years in order to lead to population-level eradication. Based on the information generated by this mathematical model, the candidate vaccines should protect individuals by raising the infectiousness threshold and secondary reduce the peak load and the duration of the infection in vaccinated individuals who become infected.

## 8. Conclusions

Vaccination could be substantially more effective than other biomedical interventions in controlling epidemics of chlamydia infection. Currently, the best public health intervention available is increasing the rate of screening and treating infected individuals. Administrating a protective vaccine to adolescents before their first sexual experience could induce a significant reduction in prevalence which could not be obtained by screening teenagers, even with a coverage of 100% [85]. Unfortunately, no protective vaccines, either fully or partially, are available although there have been many attempts to develop one. The reasons for the variability in success are still unclear but are probably a consequence of different immunization protocols and a reflection of the different protective mechanisms required for the different infections [55].
